# Conservative Nonsurgical Approach for Management of a Case of Type II Dens in Dente

**DOI:** 10.1155/2024/8843758

**Published:** 2024-01-02

**Authors:** Navin S. Agarwal, Shishir Singh, Padmini Chandrasekhar, Gaurav Kulkarni, Rajesh Podar

**Affiliations:** Department of Conservative Dentistry and Endodontics, TPCT's Terna Dental College, Nerul, Navi Mumbai, India

## Abstract

Dens in dente is a developmental dental anomaly which poses a challenge for the endodontist due to its complex pulpal anatomy. In this case report, a class 2 dens in dente was managed nonsurgically. A 32-year-old female reported with a palatal swelling and pain in the upper front region of the jaw. On examination, #7 was observed to be malformed and showed a negative pulpal sensibility test. Radiographic and CBCT analysis revealed Ohler's type 2 dens in dente with a periapical radiolucency. In the first visit, invagination was removed using diamond-coated ultrasonic tips, the canal was minimally instrumented, and premixed calcium hydroxide was injected in the canal which accidentally extruded periapically. After two weeks, a draining sinus was observed on the palatal side which when traced, lead to #7. Intracanal dressing was replaced with a mix of powdered calcium hydroxide and saline. The dressing was replaced every two weeks, and radiographs were taken, which revealed the resorption of extruded calcium hydroxide and reduction in the osseous defect. Biodentine was used to obturate the entire canal space. Subsequent follow-up radiographs till 24 months showed significant periapical healing and resorption of extruded calcium hydroxide.

## 1. Introduction

Endodontists have to face difficulties when it comes to handling various developmental dental anomalies. One such anomaly, which is clinically challenging, is dens in dente. Ploquet in 1794 reported this condition in a whale's tooth while a dentist named Socrates in 1856 described a dens in dente for the first time in human teeth [1]. Dens in dente also known as invaginatus is a dental anomaly in which a tooth is found within a tooth odontoma [2]. It is most commonly found in the maxillary lateral incisor due to the enamel organ invaginating into the dental papilla prior to calcification [3]. Being a common condition dens in dente has an incidence ranging from 0.3% to 10% [4]. The invagination initiates coronally extending up to the radicular area. The infolding of enamel into dentine creates a pocket for organic material and bacteria from the oral cavity to accumulate and grow, causing dental caries or further involvement of the pulpal space, leading to pulpal necrosis and eventually periapical pathosis [3]. There may be a number of etiological factors like trauma, infection, and improper growth pressure during odontogenesis but nothing definitive [2].

Ohler has grouped the invagination into three types [5]. A single tooth may possess more than one invagination, but each invagination may fall into a separate group.

*Type l.* A minor form of invagination, which is lined by enamel. It is confined within the crown of the tooth and does not extend beyond the level of the external cementoenamel junction.

*Type 2.* The invagination, which is enamel-lined, invades into the root but remains confined within it as a blind sac; however, there may be a communication with the pulp.

*Type 3a.* This is a complete invagination, which extends through the root and communicates with the periodontal ligament through a second foramen on the lateral aspect of the tooth. There is usually no involvement of the pulp, but it causes a significant anatomical malformation.

*Type 3b.* This is a complete invagination, which extends through the root and communicates with the periodontal ligament at the apical foramen. Often, there is no direct involvement of the pulpal anatomy, but the invagination causes significant disruption to the dental anatomy.

Treatment or endodontic outcome of such teeth with complex anatomy can be predictable only if adequate debridement and disinfection are achieved which is challenging due to the invaginated structures. Although dens invaginatus can be identified in routine 2D periapical radiograph, an adequate understanding of the complex anatomy, classification of the DI and deciding the treatment protocol for such teeth, is better achieved with cone beam computed tomography [6].

This article showcases the nonsurgical management of a case of type II dens invaginatus.

## 2. Case Report

**Figure 1:**
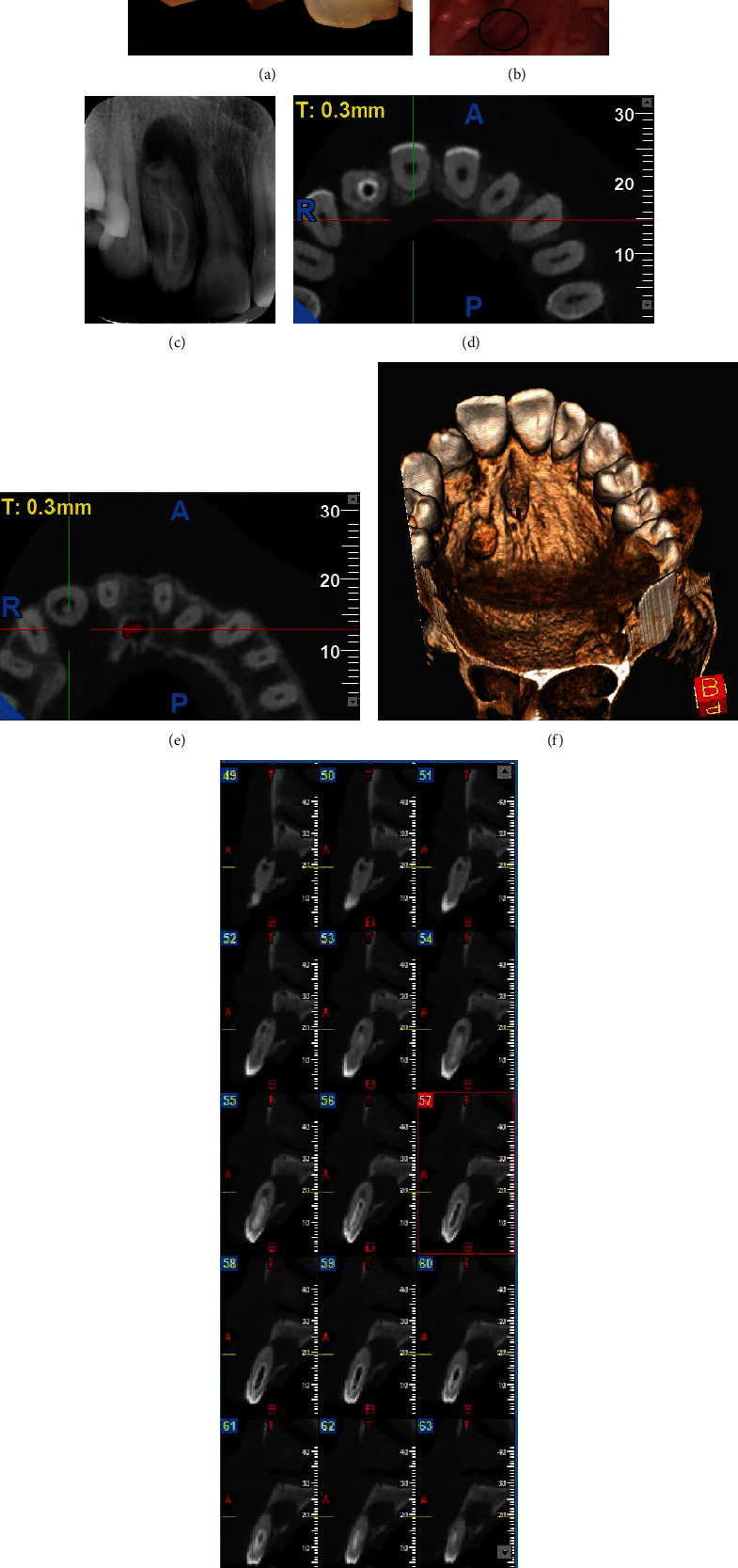
(a, b) Preoperative images of barrel-shaped #7 with palatal nondraining sinus. (c) Periapical radiograph showing complex canal anatomy and periapical lesion. (d–g) CBCT scans showing class 2 dens in dente with #7.

This case report has been written according to Preferred Reporting Items for Case reports in Endodontics (PRICE) 2020 guidelines. A 32-year-old female patient reported with a chief complaint of pain in the maxillary anterior region of the jaw along with a palatal swelling on the right side. There was no history of trauma. Clinically, the upper right lateral incisor #7 was barrel-shaped with a pit on the labioincisal aspect, it was caries-free, and there was no discoloration or tenderness on percussion and mobility (Figure 1(a)). Probing depth was normal, and a nondraining sinus was observed on the palatal aspect (Figure 1(b)). On pulp sensibility tests using Endo Ice (Hygenic, Akron, OH) and electronic pulp tester, #7 showed no response. Contralateral lateral incisor appeared normal with normal crown structure and vitality.

On radiographic evaluation, the tooth showed a radiolucent pocket with a radiopaque border extending into the root as a blind sac up to the apical third, but not reaching the apex and without connection to the periodontal ligament (Figure 1(c)). Due to a complex anatomy revealed on the IOPA, a CBCT scan was advised with #7 for detailed evaluation. On CBCT evaluation (NewTom Giano, India) #7 showed the presence of mixed density mass in root canal space, measuring 10.5 mm in length. It showed a radiodense outer structure with hypodense internal canal-like space, and the mixed density mass was confluent with the main root canal space in the apical third root region. Root resorption was seen at the apex, and a periapical osteolytic lesion measuring 8.8 mm (labiopalatally), 9.0 mm (superoinferiorly), and 8.9 mm (mesiodistally) with a dehiscence of the palatal cortical plate was observed. The clinical and radiographic evaluation led to a diagnosis of Oehler's type II DI with pulp necrosis and periapical abscess (Figures 1(d)–1(g)).

**Figure 2:**
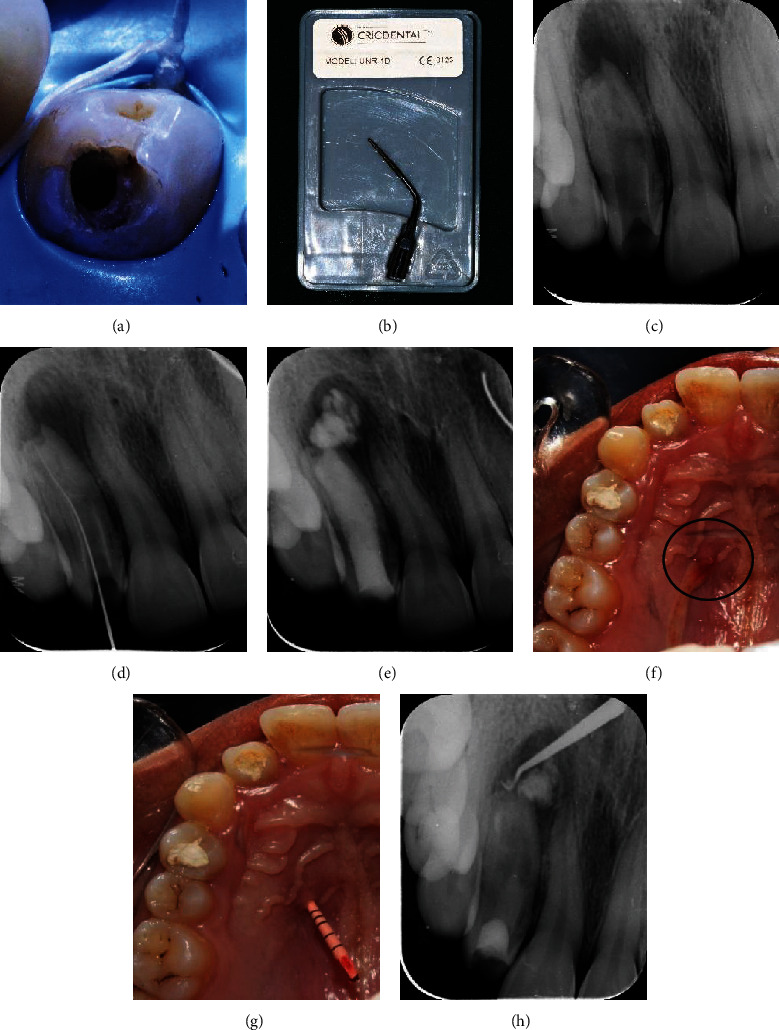
(a) Access was gained using round diamond bur. (b, c) Diamond-coated ultrasonic used to remove the invaginatus (d) working length. (e) Calcium hydroxide paste injected in the canal, leading to accidental extrusion. (f) Two weeks recall showing draining sinus on the palatal aspect. (g, h) GP tracing done after irrigation and replacement of calcium hydroxide dressing to powder and liquid form.

After explaining and discussing the treatment plan, patient's consent was obtained. In the first visit, the area was anesthetized with 1.8 mL 2% lidocaine with 1 : 80000 epinephrine (Indoco Remedies Ltd., India), and rubber dam isolation was done. Access was gained from the palatal side into the DI using round diamond point BR–41 (DIA Burs, Mani Inc., Japan) (Figure 2(a)). A DG-16 explorer (Hu-Friedy Mfg. Co., Chicago, IL, USA) was used to locate the orifices after the access was made. Pale-yellowish purulent discharge was observed which was drained, and irrigation was done with saline. The invaginated mass was removed using a diamond-coated ultrasonic tip UNR 1D (CricDental, Mumbai, India), attached to the ultrasonic handpiece (NSK, Nakanishi, Japan) with water (Figures 2(b) and 2(c)). A size 15 K-file (Mani, Tochigi, Japan) was used to determine the working length (Figure 2(d)). Minimal instrumentation of the internal canal walls was done using hand K-files. Irrigation was done using 20 mL (5.25%) sodium hypochlorite (Prime Dental Product Pvt. Ltd., India) and saline using a 27-gauge side vented needle. The canal space was dried with paper points (DiaDent, Dia-Pro T Plus, Canada), and calcium hydroxide paste (Avuecal, Dental Avenue, India) was injected into the canal, which accidentally extruded in the periapical area (Figure 2(e)). Temporization was done with 4 mm thick Tempfil–G (Shivam Dental, Jammu, India), and the patient was recalled after 2 weeks. On 2 weeks of recall, a draining sinus was observed in the palatal aspect (Figure 2(f)) which was traced radiographically using GP point (DiaDent, Dia-Pro T Plus, Canada), which lead to apex of #7 (Figures 2(g) and 2(h)). Under isolation, temporary restoration was removed with ultrasonic scaler tip (NSK, Nakanishi, Japan), and calcium hydroxide dressing was removed using saline and #50 K file (Mani, Tochigi, Japan). 3.0% sodium hypochlorite was used for irrigation with 27 gauge side vented needles and activated with U-file #15 (Mani, Tochigi, Japan). Canal was dried with paper points and intracanal dressing of calcium hydroxide powder (Deepali Dental Products Pvt. Ltd., India) mixed with saline was placed in the canal using #50 K file (Mani, Tochigi, Japan) and Machtau pluggers (Dentsply Maillefer, Ballaigues, Switzerland). Temporization was done with Tempfil-G. The similar procedure was performed every two weeks for a period of three months, till the canal was completely dry and the patient was asymptomatic.

**Figure 3:**
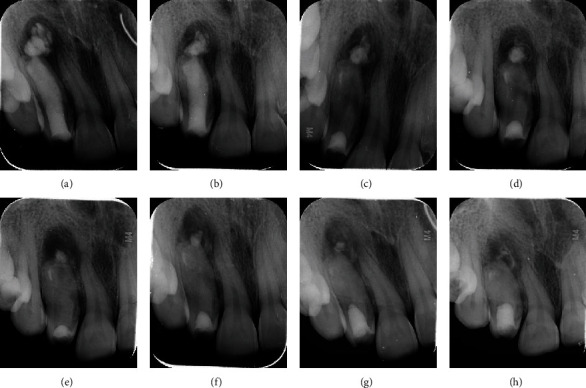
(a) Placement of injectable calcium hydroxide in the first visit. (b) Two-week follow-up. (c) Three-week follow-up. (d) Four-week follow-up. (e) Six-week follow-up. (f) Eight-week follow-up. (g) 10-week follow-up. (h) 12-week follow-up (upon every visit, calcium hydroxide dressing was replaced with freshly mixed calcium hydroxide powder in saline. Extruded calcium hydroxide resorption and periapical healing can be noted in each visit).

**Figure 4:**
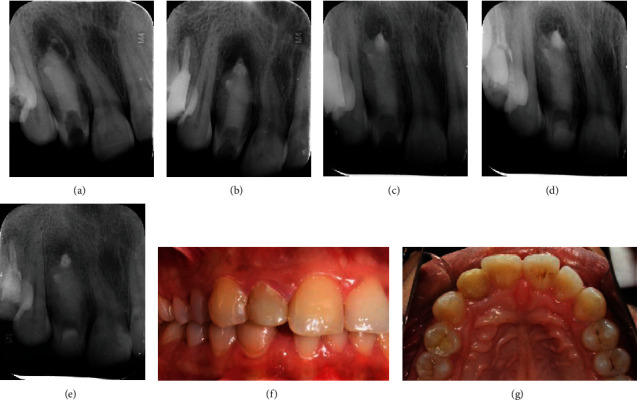
(a) Immediate post-op after obturation with Biodentine. (b) One-month follow-up postobturation. (c) 10-month follow-up postobturation (d) 12-month follow-up. (e) 24-month follow-up. (f–g) Postobturation restoration with composite resin.

Upon each visit, a periapical radiograph was taken which showed resorption of the extruded calcium hydroxide dressing and sequential osteoblastic activity and reduction in periapical radiolucency (Figures 3(a)–3(h)). After three months, the final obturation was done with Biodentine (Septodont, Saint Maur des Fosse's, France). It was mixed according to the manufacturer's instructions and placed in sections with the help of Machtou plugger size #4 which was kept 2 mm short of the apex for void-free obturation and to avoid overextension. Full length of the canal up to the cementoenamel junction was filled with Biodentine, and temporization was done with glass ionomer cement (Figure 4(a)).

On 1-month postobturation recall, the patient was asymptomatic, and the periapical area showed signs of healing with further resorption of extruded calcium hydroxide (Figure 4(b)). On 10 months and 12 months of recall, the glass ionomer cement was completely intact with no signs of microleakage, and the extruded calcium hydroxide had resorbed with good osteoblastic activity and periapical healing (Figures 4(c) and 4(d)). Glass ionomer cement was reduced to 2 mm as an intraorifice barrier, and a permanent restoration was done using composite resin (Tetric N Ceram, Ivoclar Vivadent, Liechtenstein), followed by finishing and polishing (Figures 4(f) and 4(g)). 24-month follow-up shows complete periapical healing with good osteoblastic activity and an intact postobturation restoration (Figure 4(e)).

## 3. Discussion

Dens invaginatus is a developmental dental anomaly which is formed during tooth development, when the enamel organ is invaginated into dental papilla resulting in the formation of a pit lined by enamel and dentin invaginating the pulp [6]. The pit which is formed provides an area for the stagnation of organic matter and eventually provides hospitable environment for the growth of microorganisms [7]. The enamel lining of the DI is generally malformed and contains fine canals which provide a pathway for the spread of microbes from the infected pits to the surrounding vital pulp tissue of the tooth, eventually leading to necrosis [6].

CBCT helped in revealing the type of DI, its extension, surrounding complex pulpal anatomy, and the periapical lesion. The initial clinical and radiographic examination revealed the complications of the endodontic treatment pertaining to such complex anatomy where much of the pulpal space was occupied by the invaginated mass, which might hinder the complete debridement and disinfection of the entire pulpal space. Treatment procedure was decided after CBCT evaluation which revealed that the invaginatus was not attached to the radicular dentin and careful removal of the DI was decided. Although retaining the DI might increase the root strength, the complete debridement and adequate endodontic treatment of the DI and the pulp separately become complex, affecting the prognosis [8]. As in this case, the invagination was close to the apical third, and the diamond-coated ultrasonic tip was used for the removal. This helped the inadequate removal of the invagination without compromising the radicular dentin.

Calcium hydroxide paste was used as an intracanal medicament which was injected in the canal space using a syringe delivery system leading to accidental extrusion in the periapical area due to the pressure of the syringe and lack of apical stop. It was replaced after 2 weeks with manually mixed powdered calcium hydroxide with saline and placed using K files and pluggers to avoid extrusion. De Moor et al. [9] found that, although the calcium hydroxide pastes were highly alkaline, overextensions into periradicular lesions, in general, result in mild and transient tissue reactions, where extensive calcium hydroxide extrusion may require more than 6 months to repair. A deliberate overextension is avoidable. In this case, on further recalls, resorption of extruded calcium hydroxide and periapical healing was seen. After 12 weeks of clinical and radiographic examination, obturation of the entire canal was done with Biodentine, and temporization was done with glass ionomer cement. Biodentine is a calcium silicate-based material with good biocompatibility, and studies have shown improved fracture resistance of tooth obturated with bioceramic material as it reinforces the radicular dentine [10–13]; hence, in this case, Biodentine was used as the radicular dentine thickness was less. On subsequent follow-ups, further resorption of extruded calcium hydroxide was observed which remained negligible on 12-month follow-up, with reduction in periapical radiolucency and good healing radiographically. Direct composite restoration was preferred, as the remaining pericervical dentin thickness was inadequate and tooth preparation to receive an indirect restoration would have further compromised the tooth structure. On a 24-month follow-up, an intact postobturation restoration clinically and complete periapical healing were observed radiographically.

## 4. Conclusion

Earlier the nonsurgical treatment of dens invaginatus with such complex, anatomy and periapical lesion was not considered feasible. However, advent of advanced technology like 3D imaging aids in a better understanding of complex anatomy which facilitates the treatment planning. Also, the usage of instruments like ultrasonics in conjunction with bioceramic materials helps in minimal tooth structure loss and maintaining the biological and mechanical integrity of the endodontically treated tooth. And hence, such complex treatment could be feasible nonsurgically with favourable outcome.
